# Supplementary material for: Contrasted Epidemiological Patterns of West Nile Virus Lineages 1 and 2 Infections in France from 2015 to 2019
Source: Pathogens. 2020 Oct 30;9(11):908. doi: 10.3390/pathogens9110908 (PMC7692118; doi:10.3390/pathogens9110908)
Supplement: Supplementary file 1 [file pathogens-09-00908-s001.pdf]

**Table S1**: Table presenting percentage homology in nucleotidic (nt, upper part) and in amino acid (aa, lower part) WNV sequences[1].

| | WNV-Akela /France/2015, MT863559 | WNV-405/04 /France/2004, DQ786572 | WNV-PaAn001 /France/2000, AY268132 | WNV-7025 /France/2018, MT863561 | WNV-Cremona4 /Italy/2014, KP789957 |
|---|---|---|---|---|---|
| WNV-Akela /France/2015, MT863559 | | 98,55% (10760/10918) | 98,31% (10737/10922) | 79,72% (8749/10975) | 79,79% (8756/10974) |
| WNV-405/04 /France/2004, DQ786572 | 99,59 % (3420/3434) | | 98,54% (10779/10938) | 79,65% (8752/10988) | 79,72% (8759/10987) |
| WNV-PaAn001 /France/2000, AY268132 | 99,74 % (3425/3434) | 99,62% (3421/3434) | | 79,54% (8275/10403) | 79,62% (8282/10402) |
| WNV-7025 /France/2018, MT863561 | 93,83% (3223/3435) | 93,80% (3222/3435) | 93,80% (3222/3435) | | 99,76% (10969/10995) |
| WNV-Cremona4 /Italy/2014, KP789957 | 93,97% (3228/3435) | 93,94% (3227/3435) | 93,94% (3227/3435) | 99,76% (3427/3435) | |

nt homology

aa homology

[1]Whole-genome sequences of recent WNV isolates (WNV-Akela /France/2015 and WNV-7025 /France/2018) were compared to French and Italian complete genome sequences. Pairwise nucleotide and aa sequence alignment were performed with blastn and blastp respectively (https://blast.ncbi.nlm.nih.gov/).

**Table S2**: Amino acid substitutions identified between WNV-Akela/France/2015 and older French lineage 1 isolates

| AA position in the WNV polyprotein | Gene | WNV-Akela /France/2015, MT863559 | WNV-PaAn001 /France/2000, AY268132 | WNV-405/04 /France/2004, DQ786572 |
|---|---|---|---|---|
| 100 | C | S | S | L |
| 119 | C | A | A | T |
| 158 | prM | I | I | T |
| 383 | E | K | R | R |
| 443 | E | G | G | R |
| 602 | E | L | L | F |
| 836 | NS1 | I | V | I |
| 929 | NS1 | P | P | S |
| 932 | NS1 | K | K | R |
| 1271 | NS2A | E | G | E |
| 1362 | NS2A | V | A | A |
| 1749 | NS3 | H | H | Q |
| 2008 | NS3 | N | S | N |
| 2288 | NS4B | R | S | S |
| 2532 | NS5 | R | K | K |
| 2572 | NS5 | K | R | R |
| 2902 | NS5 | Y | Y | H |
| 3209 | NS5 | T | I | T |
| Non synonymous mutations | | | 9 | 14 |

**Table S3**: Amino acid substitutions identified between lineage 2 WNV-7025/France/2018 and the closely genetically related WNV-Cremona4/Italy/2014

| AA position in the WNV polyprotein | Gene | WNV-Cremona4 /Italy/2014, KP789957 | WNV-7025 /France/2018, MT863561 |
|---|---|---|---|
| 207 | prM | T | P |
| 345 | E | D | V |
| 883 | NS1 | K | E |
| 1340 | NS2A | K | X |
| 1726 | NS3 | V | I |
| 2557 | NS5 | R | K |
| 2801 | NS5 | F | L |
| 3322 | NS5 | V | A |
| non-synonymous mutations | | 8 | |
